# Caecal Microbiota of Experimentally *Campylobacter jejuni-*Infected Chickens at Different Ages

**DOI:** 10.3389/fmicb.2019.02303

**Published:** 2019-10-10

**Authors:** Julia Hankel, Klaus Jung, Henrike Kuder, Birgit Keller, Christoph Keller, Eric Galvez, Till Strowig, Christian Visscher

**Affiliations:** ^1^Institute for Animal Nutrition, University of Veterinary Medicine Hannover, Hanover, Germany; ^2^Institute for Animal Breeding and Genetics, University of Veterinary Medicine Hannover, Hanover, Germany; ^3^Boehringer Ingelheim Veterinary Research Center GmbH & Co. KG, Hanover, Germany; ^4^Helmholtz Center for Infection Research, Braunschweig, Germany

**Keywords:** foodborne pathogen, *Campylobacter jejuni*, 16S rRNA, gut microbiota, volatile fatty acids

## Abstract

*Campylobacter jejuni* is the most common bacterial cause of foodborne zoonosis in the European Union. Infections are often linked to the consumption and handling of poultry meat. The aim of the present study was to investigate the caecal microbiota of birds infected with *C. jejuni* at different ages. Therefore, a total of 180 birds of the laying hybrid Lohmann Brown-Classic were housed in 12 subgroups of 15 animals each in three performed repetitions. Three birds per subgroup were experimentally infected with *C. jejuni* at an age of about 21 days and about 78 days (4.46 ± 0.35 log_10_ CFU/bird). Twenty-one days after experimental infection, microbiome studies were performed on 72 caecal samples of dissected birds (three primary infected and three further birds/subgroup). Amplification within the hypervariable region V 4 of the 16S rRNA gene was performed and sequenced with the Illumina MiSeq platform. Statistical analyses were performed using SAS^®^ Enterprise Guide^®^ (version 7.1) and R (version 3.5.2). Both factors, the experimental replication (*p* < 0.001) and the chickens’ age at infection (*p* < 0.001) contributed significantly to the differences in microbial composition of the caecal samples. The factor experimental replication explained 24% of the sample’s variability, whereas the factor age at infection explained 14% thereof. Twelve of 32 families showed a significantly different count profile between the two age groups, whereby strongest differences were seen for seven families, among them the family *Campylobacteraceae* (adjusted *p* = 0.003). The strongest difference between age groups was seen for a bacterial species that is assigned to the genus *Turicibacter* which in turn belongs to the family *Erysipelotrichaceae* (adjusted *p* < 0.0001). Correlation analyses revealed a common relationship in both chicken ages at infection between the absolute abundance of *Campylobacteraceae* and *Alcaligenaceae*, which consists of the genus *Parasutterella.* In general, concentrations of particular volatile fatty acids (VFA) demonstrated a negative correlation to absolute abundance of *Campylobacteraceae*, whereby the strongest link was seen for n-butyrate (−0.51141; *p* < 0.0001). Despite performing consecutive repetitions, the factor experimental replication contributed more to the differences of microbial composition in comparison to the factor age at infection.

## Introduction

More than 100 years ago, chickens were already recognised as an important source of zoonotic infection (Higgins, 1898). Nowadays, the zoonotic pathogen *Campylobacter jejuni* is the most common bacterial cause of foodborne zoonosis in the European Union, with infections often being linked to the consumption and handling of poultry meat (Efsa, 2017). Intestinal colonisation of chickens results in faecal contamination of the carcasses during the slaughtering process (Rosenquist et al., 2006; Guerin et al., 2010). One approach to eliminating *C*. *jejuni* from the food chain is to prevent the colonisation of broiler chickens (Newell and Fearnley, 2003). The extent of contamination shows a positive correlation between the number of *Campylobacter* present in the caecal content and the number of bacteria on the carcasses and cut products (Rosenquist et al., 2006; Reich et al., 2008). Therefore, reduced prevalence and *Campylobacter* load are strived for at production level prior to slaughter (Hermans et al., 2011).

Investigations have already demonstrated that with advanced age of the birds, the levels of *Campylobacter* excretion are lower compared to younger birds (Glünder, 1995; Colles et al., 2009; Hankel et al., 2018). Hankel et al. (2018) observed higher *C*. *jejuni* counts in caecal contents of young chickens 21 days after experimental infection with *C*. *jejuni* in comparison to those in old ones. Of the investigated influencing factors genetics, age and diet composition, the factor age was identified as the most influential variable and the only variable affecting caecal *Campylobacter* counts, with significant differences of almost two log steps between young and old birds (log_10_ CFU/g: 8.57 ± 0.46 vs. 6.66 ± 1.43). These findings might lead to the assumption that a higher age at infection is one of the greatest influencing factors on *C. jejuni* load in the chicken intestine. It is of great interest to make further investigations concerning the characteristics of the environment in which the bacterium lives in order to detect possible influences. Despite the fact that the chicken gut microbiome is being increasingly characterised by means of modern sequencing approaches, little is known about the factors influencing its modulation (Thibodeau et al., 2015) or the abilities of most species present in the caecum (Qu et al., 2008). Commensal bacteria benefit the host by their ability to competitively exclude other bacteria from colonising the intestine (Brisbin et al., 2008; Buffie and Pamer, 2013). Investigations with laying hybrid lines offer the opportunity to examine a possible influence of the chicken’s age on *Campylobacter*, as they are also marketed with advanced age. Regarding the marketing standards for poultry meat (COMMISSION REGULATION (EC) No. 543/2008), male chickens of laying strains have to be slaughtered at an age of at least 90 days to be marketed as young cocks. Alternatively, chickens of less than 650 g carcass weight (expressed without giblets, head, and feet) can be marketed as poussin, also called coquelet. The aim of the present study was to examine under standardised conditions the bacterial composition in the caecum of *C. jejuni*-infected chickens at different ages in three consecutive trials. We hypothesised that possible large variations in microbiota due to the chickens’ age would affect the abundance of *Campylobacteraceae* in caecal contents of chickens that were experimentally infected with *C. jejuni* under equal standardised procedures. Deeper knowledge concerning the susceptibility of chickens to members of the *Campylobacteraceae* family are of great interest to take preventive measures against this zoonotic pathogen in order to increase food safety in the poultry industry.

## Materials and Methods

The study was carried out with the aim of clarifying the question as to why a *Campylobacter* infection, susceptibility and excretion are age dependent. Against this background, caecal microbiota and the concentrations of bacterial fermentation products of experimentally *C. jejuni*-infected chickens at different ages were examined.

### Experimental Design, Animals Housing and Sampling

The birds of the breed Lohmann Brown-Classic were supplied as day-old chicks from the same hatchery (Lohmann Tierzucht GmbH, Cuxhaven, Germany). Only male birds were used for the experiments.

**FIGURE 1:**
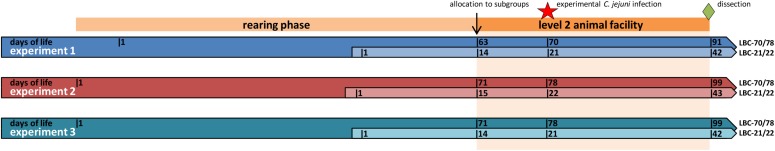
Schedule of the experiments with actions in chickens of different ages at infection (LBC-21/22, Lohmann Brown-Classic, infection at the age of 21/22 days; LBC-70/78, Lohmann Brown-Classic, infection at the age of 70/78 days).

The investigations took place in three independent repetitions (experiments 1–3, *r* = 3) where in total 180 birds were reared under the same conditions (Figure 1).

During the rearing phase, all birds were reared in identical floor pens. Pens were littered with wood shavings. The duration of the rearing phase was variable due to the study’s objective to infect a part of the birds at an advanced age. For this reason, half of the birds were reared for about 2 weeks (LBC-21/22), whereas the other half of the birds were reared for 9 weeks in experiment 1 and 10 weeks in experiments 2 and 3 (LBC-70/78) before being transferred from the rearing to the infection unit of a level 2 animal facility. The birds were kept for a further 4 weeks on solid floor pens. Pens were littered with wood shavings (1 kg/m^2^). Stocking density amounted to a maximum of 30 kg per square metre. The birds were maintained on a 16 L:8 D light schedule during the whole experiment. Each age group was randomly assigned to two subgroups per experiment, each made up of 15 birds, so that each age group consisted of 30 birds per experiment. One week after transferring the animals to the infection unit, an experimental challenge with *C*. *jejuni* took place in all subgroups. In accordance with Hankel et al. (2018), three of 15 broilers of each subgroup (seeder birds) were administered orally with a 1 mL inoculum of *C*. *jejuni* (4.46 ± 0.35 log_10_ CFU/mL). Qualitative detection of *C*. *jejuni* in cloacal swabs of all birds (days 2, 4, 7, 14, and 21 after inoculation) and quantitative detection of *C*. *jejuni* in excreta samples of seeder birds (days 2, 11, and 17 after inoculation) and in caecal samples of all birds at dissection were performed during the experimental period in order to examine prevalence and excretion of *C. jejuni*. The results were already published in Hankel et al. (2018). Three weeks after the experimental challenge with *C*. *jejuni*, all chickens were dissected (LBC-21/22: at an age of 42 days in experiments 1 and 3 and 43 days in experiment 2; LBC-70/78: at an age of 91 days in experiment 1 and 98 days in experiments 2 and 3). Anaesthesia and killing of birds were carried out in accordance with Annex 2 [to paragraph 2 (2)] of the Regulations on the Welfare of Animals Used for Experiments or for Other Scientific Purposes (TierSchVersV). Anaesthesia was performed by head stroke. After bleeding, caecal contents were removed under sterile conditions and placed in reaction vessels. In addition, caecal contents of the seeder birds and three further birds/subgroup were immediately frozen and stored at −80°C for further microbiota analyses. Unfortunately, no caecal chyme was available from one bird in the LBC-21/22 group. One further caecal sample of one bird in the LBC-70/78 group was stored instead.

### Feeding Regime, Diet, and Performance Parameters

The birds were given conventional complete diets based on wheat, soybean meal, corn and rape cake, which were supplemented by whole wheat, coccidiostats and NSP-degrading enzymes. The diets were produced and delivered by Best 3 Geflügelernährung GmbH (Twistringen, Germany). The diets were designed in accordance with the recommendations for energy and nutrient supply of the laying hens and fowls (broilers) of the Committee for Needs Standards of the Society of Nutrition Physiology (GfE, 1999). Energy content and nutrient composition of the diets can be taken from Hankel et al. (2018).

The rearing phase was divided into a 1-week starter phase during which a conventional pelleted starter diet was offered (starter) and a subsequent 7-day (LBC-21/22), or 56/64-day phase (LBC-70/78) with a commercially available pelleted grower diet (grower). The finisher diet was offered beginning from week 3, or beginning from week 10/11 until dissection. All diets were offered *ad libitum*. At level 2 animal facility water was offered *ad libitum* in drinking lines equipped with Top Nipples and drinking cups (Big Dutchman International GmbH, Vechta-Calveslage, Germany) The water was treated with 0.3 mg free chlorine/L (Virbac Clean Pipe, VIRBAC Tierarzneimittel GmbH, Bad Oldesloe, Germany).

At the day the animals were transferred to the infection unit and at the end of the trial the individual body weight of birds was determined as well as the feed intake being measured at subgroup level. The average daily feed intake/bird and the feed conversion ratio (FCR) at subgroup level could be calculated.

### Analyses

#### Chemical Analyses

Caecal chyme was homogenised and the volatile fatty acids (VFA) concentration was measured by gas chromatography (610 Series, Unicam, Kassel, Germany). The samples were mixed with an internal standard (10 mL of formic acid [89%] and 0.1 mL of 4-methylvaleric acid). The mixture was centrifuged and afterward subjected to gas chromatography with a column temperature of 155°C (injector: 175°C, detector: 180°C).

#### Bacteriological Analyses

Qualitative and quantitative detection of *C*. *jejuni* was done in caecal samples of all 180 birds at dissection.

The qualitative bacteriological examination was based on the DIN EN ISO 10272–1:2006 in accordance with § 64 LFBG. Pre-enrichment was performed in Bolton Boullion, a liquid selective nutrient medium. Samples that were to be examined were incubated in a one-to-nine ratio (sample:Bolton Boullion) in sterile 5 mL tubes mounted with a vent cap (Sarstedt AG & Co., Nuembrecht, Germany) for 4 h at 37°C followed by 44 ± 4 h at 41.5°C in a microaerobic atmosphere (oxygen content of 5 ± 2%, carbon dioxide content of 10± 3%). Microaerobic atmosphere was created in a CO_2_ incubator with O_2_ control (CB 160, BINDER GmbH, Tuttlingen, Germany). After enrichment, sterile 10 μL inoculation loops were used to streak the samples onto two solid selective culture media (mCCD agar and Karmali agar; Oxoid Germany GmbH, Wesel, Germany) and afterward incubated again for 44 ± 4 h at 41.5°C in a microaerophilic atmosphere. To confirm the presence of *Campylobacter* individual colonies were analysed by phase contrast microscopy (Distelkamp-Electronic, Kaiserslautern, Germany) and biochemical methods (API Campy, bioMérieux SA, Marcy- l‘Etoile, France).

For quantitative bacteriological examination 0.5 g sample material was diluted. A ten-fold dilution series was made with phosphate buffered saline (PBS, Phosphate Buffered Saline, Oxoid Germany GmbH, Wesel, Germany). In duplicate, 100 μL of each dilution was plated onto mCCD agar (Oxoid Germany GmbH, Wesel, Germany). After incubation in a microaerophilic atmosphere for 44 ± 4 h at 41.5°C, the colonies were counted and an average value from the two duplicate experiments was taken for calculating the CFU/g intestinal content.

#### 16S rRNA Analyses

A total of 72 samples were included in the study. All caecal samples of the 72 birds included in the study were qualitatively detected as *C. jejuni* positive. Samples were stored at −80°C until simultaneous analysis. Mixer mill (Retsch MM 400, Haan, Germany) was used to homogenise the chyme for 1 min before DNA-extraction was done on an automated liquid handler (Microlab Star, Hamilton Germany GmbH, Gräfelfing, Germany) based on the DNeasy Blood&Tissue Kit (Qiagen, Hilden, Germany). An additional purification step (Kit: BS 365, BioBasic, Ontario, Canada) was performed before the hypervariable region V 4 of the 16S rRNA gene was amplified using primer F515/R806 in accordance with previously described protocols (Caporaso et al., 2011). Sequencing the amplicons was done on the Illumina MiSeq platform (PE250) and the Usearch8.1 software package^1^ was used to assemble, quality control and cluster obtained reads. Reads were merged using -fastq_mergepairs –with fastq_maxdiffs 30. Chimeric sequences were identified and removed using cluster_otus (-otu_radius_pct 3) and the Uchime command included in the Usearch8.1 workflow. Quality filtering was set up with fastq_filter (-fastq_maxee 1); minimum read length, 200 bp. Reads were clustered into 97% ID operational taxonomic units (OTUs). The OTU clusters and representative sequences were determined using the UPARSE algorithm (Edgar, 2013). Taxonomy assignment was done with the help of Silva database v128 (Quast et al., 2013) and the RDP Classifier (Wang et al., 2007) with a bootstrap confidence cutoff of 70%.

### Statistical Analyses

For the statistical evaluation of counts of *C*. *jejuni* in caecal content samples determined via quantitative bacteriological examination, the data were logarithmised. Statistical analyses were performed using SAS (version 7.1, SAS Institute Inc., Cary, NC, United States). The performance data and counts of *C*. *jejuni* in the caecal content were analysed with respect to the factor age at infection by one-way analysis of variance (ANOVA) for independent samples. All statements of statistical significance were based upon *p*-values smaller than 0.05.

Statistical analyses of microbiota were performed using R (version 3.5)^2^ with the R-package “phyloseq” (version 1.24.4) (McMurdie and Holmes, 2013). Permutational multivariate analysis of variance (PERMANOVA) on Bray-Curtis distances was used to identify factors contributing to the differences in microbial composition of the samples. The Bray-Curtis dissimilarity matrix was used to compare community dissimilarity based on abundance of OTUs, whereas the Jaccard distance was additionally used to compare community dissimilarity based on presence/absence of OTUs. Sample diversity was measured with the species richness estimators Observed Species and Chao 1 index, whereas the Shannon index characterises species diversity accounting for abundance and evenness of the species. To find taxa with significantly different abundance between age groups, counts were normalised and compared using the R-package DESeq (version 1.24) which uses tests based on the negative binomial distribution (Love et al., 2014). To identify phyla with significantly different abundance profile the rotation test implemented in the R-package “limma” was used which acts in a similar manner to a permutation test (Wu et al., 2010). In the taxa and phylum specific analyses, experiment was included in the models as independent factor. *P*-values from these tests were either adjusted by the Bonferroni-Holm (phyla) or Benjamini and Hochberg (“BH”, taxa) method to control either for a family-wise error rate or the false discovery rate of 5%, respectively. The Spearman’s rank correlation was performed to measure the strength of association between absolute abundance data of *Campylobacteraceae* to other bacterial families within the caecal microbiota as well as to the concentrations of VFAs in the caecal content.

## Results

The general health of each animal was checked at least twice a day. The experiments ran without complications. No animal losses occurred after the rearing phase.

### Performance Data

At dissection, significant differences in bodyweight as a function of age were present (LBC-70/78: 1834 g ± 137 > LBC-21/22: 595 g ± 59.0, *p* < 0.0001). The FCR (kg/kg, in the last 4 weeks before dissection) was significantly higher for LBC-70/78 (3.67 ± 0.28) in comparison to LBC-21/22 (2.12 ± 0.07, *p* < 0.0001).

### Caecal Campylobacter Counts

Except for one bird (LBC-70/78), qualitative bacteriological examination revealed all caecal samples as *C. jejuni* positive.

*Campylobacter* counts in the caecum differed significantly depending on the age of the chickens (*p* < 0.0001). The LBC-70/78 chickens had significantly lower numbers of *C*. *jejuni* in the caecum (6.66 ± 1.43 log_10_ CFU/g) in comparison to the LBC-21/22 chickens. Both groups differed by almost two log steps (LBC-21/22: 8.57 ± 0.46 log_10_ CFU/g).

### Intestinal Microbiota

The dataset contained 1,034,213 reads (average number of reads: 14,364; range: 4,170–87,441) mapped to 216 OTUs.

#### Alpha and Beta Diversity

Despite carrying out the experiments under the same conditions, the PERMANOVA test on Bray-Curtis dissimilarities indicated that both the experimental replication (*p* < 0.001) and the age at infection (*p* < 0.001) contributed significantly to the differences in microbial composition of the caecal samples. The factor experimental replication explained 24% of the sample’s variability, whereas the factor age at infection explained 14% thereof. The fact that birds were primary infected (seeder birds) or became infected during *C. jejuni* spread did not contribute to the differences in microbial composition of the samples, neither in LBC-21/22 samples (*p* = 0.790) nor in LBC-70/78 samples (*p* = 0.804).

**FIGURE 2:**
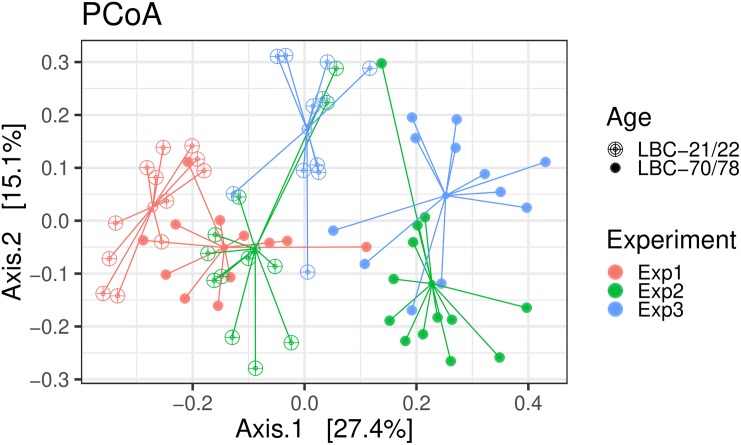
Bray-Curtis dissimilarity-based principal coordinate analysis (PCoA) was performed on caecal samples collected in experiments 1–3. Each point represents a different bird infected at different age [LBC-21/22, Lohmann Brown-Classic, infection at the age of 21/22 days (open points); LBC-70/78, Lohmann Brown-Classic, infection at the age of 70/78 days (filled points)]; coloured lines connect birds of one experiment (Exp 1, Experiment 1; Exp 2, Experiment 2; Exp3, Experiment 3).

Ordination was performed using Bray-Curtis dissimilarity-based principal coordinate analysis (PCoA, Figure 2) also provided in the R-package “phyloseq”. Dissimilarity matrix (Table 1) showed the highest community dissimilarity between Exp 1 and Exp 3. Jaccard dissimilarity indicated that for all experiments, both age groups shared half of the OTUs (50.2%, Table 2).

**TABLE 1:** Community dissimilarity of microbiota in caecal contents of birds collected in three consecutive and similarly performed trials based on Bray-Curtis and Jaccard distance.

Bray-Curtis		Exp1	Exp2
	Exp2	0.3658507	
	Exp3	0.4537453	0.2774941
Jaccard		Exp1	Exp2
	Exp2	0.5357112	
	Exp3	0.6242432	0.4344350

*Community dissimilarity with a range from 0 (similar) to 1 (dissimilar); Exp1, Experiment 1; Exp2, Experiment 2; Exp3, Experiment 3.*

**TABLE 2:** Community dissimilarity of microbiota in caecal contents of birds infected at different ages based on Bray-Curtis and Jaccard distance.

Bray-Curtis		LBC-21/22

	LBC-70/78	0.3317109

Jaccard		LBC-21/22

	LBC-70/78	0.4981725

*Community dissimilarity with a range from 0 (similar) to 1 (dissimilar); Exp1, Experiment 1; Exp2, Experiment 2; Exp3, Experiment 3; LBC-21/22, Lohmann Brown-Classic, infection at the age of 21/22 days; LBC-70/78, Lohmann Brown-Classic, infection at the age of 70/78 days.*

**FIGURE 3:**
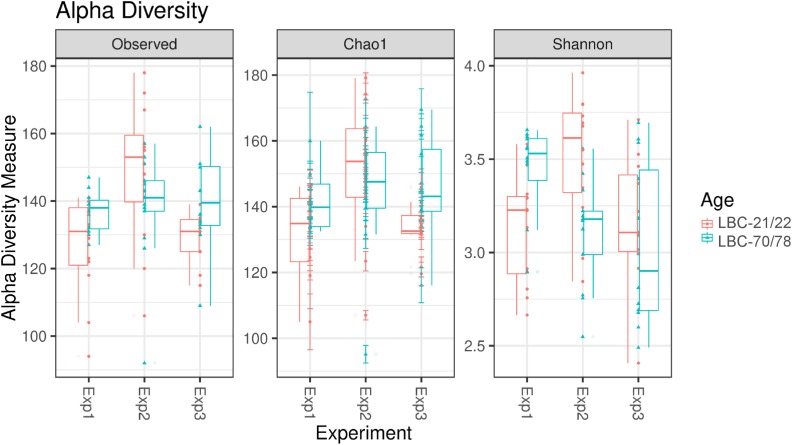
Alpha diversity in samples from caecal content depending on experiment and age at infection. Box-plots showing alpha diversity in samples using the Observed Species index, the Chao1 index and Shannon index (Exp1, Experiment 1; Exp2, Experiment 2; Exp3, Experiment 3; LBC-21/22, Lohmann Brown-Classic, infection at the age of 21/22 days; LBC-70/78, Lohmann Brown-Classic, infection at the age of 70/78 days).

Alpha diversity of microbiota in caecal content of chickens of different ages at infection in the three consecutive experiments are pictured as box-plots using the indices Observed Species, Chao1, and Shannon (Figure 3). Table 3 shows statistical analyses of alpha diversity indices Observed Species, Chao 1 and Shannon for the factors chickens’ ages at infection and experiment. Irrespective of the factor experimental replication, no significant differences were found for all measured alpha diversity indices between chickens of different ages at infection.

**TABLE 3:** Comparison of alpha-diversity indices of the microflora in caecal contents of chickens.

**Index**	**Factor**	***p*-value**
Observed	Age at infection	0.28014
	Experimental replication	0.02147
	Age at infection: Experimental replication	0.01617
Chao 1	Age at infection	0.14196
	Experimental replication	0.04055
	Age at infection: Experimental replication	0.04862
Shannon	Age at infection	0.27704
	Experimental replication	0.08584
	Age at infection: Experimental replication	0.00326

#### Relative Abundance per Sample and Age Effects on the Phylum and OTU Level

**FIGURE 4:**
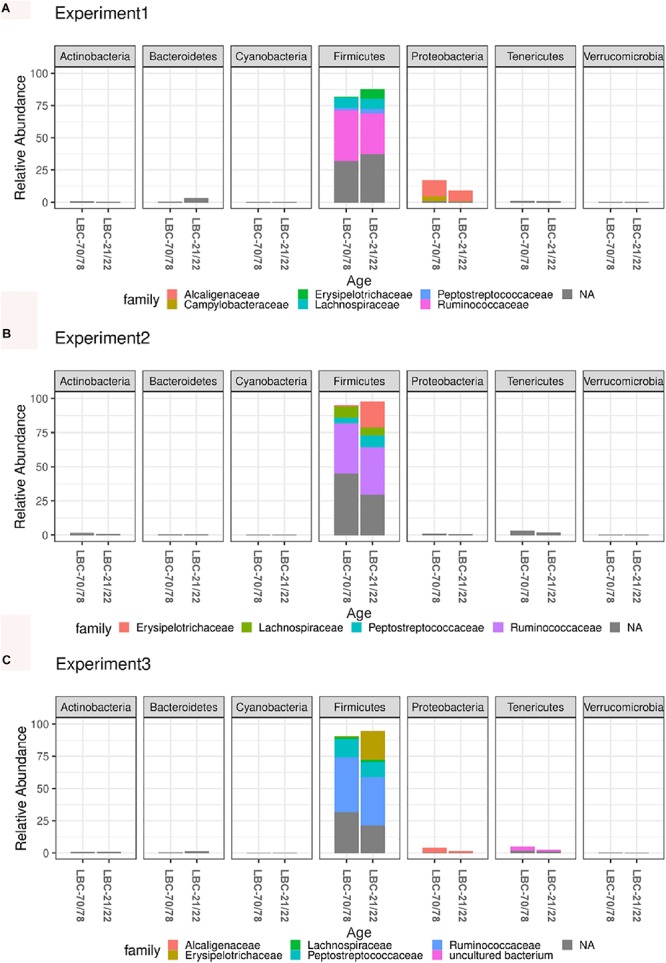
Microbiota composition analysis using 16S rRNA sequencing in caecal contents of different aged chickens at time of infection. Relative abundance of the 11 most abundant OTUs belonging to bacterial families within different phyla in experiment 1 **(A)**. Relative abundance of the 11 most abundant OTUs belonging to bacterial families within different phyla in experiment 2 **(B)**. Relative abundance of the 11 most abundant OTUs belonging to bacterial families within different phyla in experiment 3 **(C)**. LBC-21/22, Lohmann Brown-Classic, infection at the age of 21/22 days; LBC-70/78, Lohmann Brown-Classic, infection at the age of 70/78 days; NA, OTUs without genus-level taxonomic assignment.

Independent of the chickens’ age at infection and experiment, the caecal microbiota was dominated at phylum level by *Firmicutes* (91.1%) and *Proteobacteria* (5.02%), followed by *Tenericutes* (2.25%) and *Bacteroides* (1.00%). Relative abundance of bacterial phyla within all samples is shown in Supplementary Figure S1. Older animals showed consistently higher values for relative abundance of *Firmicutes* in all experiments. Relative abundance of *Firmicutes* reached highest values in experiment 2 (LBC-21/22: 94.7%; LBC-70/78: 97.6% of total microbiota), while lowest relative abundance of *Firmicutes* was found in experiment 1 (LBC-21/22: 82.9%; LBC-70/78: 87.4% of total microbiota). *Campylobacteraceae* could be found within the most abundant eleven OTUs in experiment 1. For this reason, the default presentation of the most abundant ten OTUs was extended by one OTU (Figure 4). Experiment 1 showed a conspicuously higher relative abundance of *Proteobacteria* in comparison to the other experiments. *Proteobacteria* mainly consisted of the families *Alcaligenaceae* and *Campylobacteraceae* in experiment 1 and *Alcaligenaceae* in experiment 3, while in experiment 2, this family was not present among the 11 most abundant OTUs (Figure 4). The 11 most abundant OTUs in experiment 2 all belonged within the phylum *Firmicutes* to the families *Erysipelotrichaceae, Lachnospiraceae*, *Peptostreptococcaceae*, and *Ruminococcaceae*. OTUs belonging to these four families were found among the 11 most abundant ones in all experiments.

**FIGURE 5:**
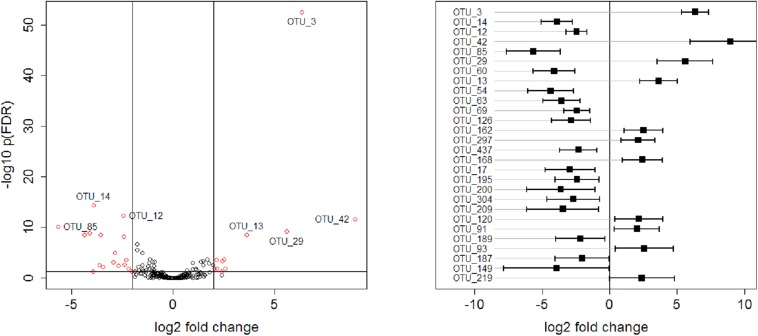
**Left**: Volcano plot representing –log10 FDR-adjusted *p*-values versus log2 fold changes for all 216 OTUs. **Right**: log2 fold changes of 28 OTUs, selected with a criterion of FDR-adjusted *p*-values < 0.05 and absolute log2 fold change >2. OTUs are ordered according to *p*-values, with the OTU_3 having the smallest *p*-value. Confidence intervals were calculated according to Jung et al. (2011).

Global tests on normalised counts on each phylum yielded five (of seven) phyla that showed a significantly different count profile between the two age groups (Supplementary Table S1). The strongest difference between age groups was seen between *Firmicutes* (adjusted *p* = 0.0007), whereas *Cyanobacteria* and *Verrucomicrobia* were not significant. Global tests on normalised counts yielded twelve (of 32) families that showed a significantly different count profile between the two age groups (Supplementary Table S2). The strongest difference between age groups was seen between *Campylobacteraceae*, *Enterococcaceae*, *Erysipelotrichaceae*, *Lachnospiraceae*, *Lactobacillaceae*, *Peptostreptococcaceae*, and *Ruminococcaceae* (adjusted *p* = 0.00259974). At the species level, 28 of 216 OTUs showed a significantly (plus an additional log fold change criterion of ± 2) different abundance between the two age groups, where 11 of these OTUs were more enriched in the older animals and 17 more enriched in the younger animals. Log fold changes for these 28 OTUs are shown in Supplementary Table S3 and plotted in Figure 5. OTU_3 had the smallest *p*-value, followed by OTU_14.

#### Correlation Analysis

Spearman’s rank correlation coefficient was used to quantify the strength of association and the direction of the relationship between *Campylobacteraceae* and other taxa at the taxonomic level family (Table 4). There was a positive Spearman’s rank correlation between absolute abundances of *Campylobacteraceae* and *Alcaligenaceae* in caecal contents of young (Spearman’s rank correlation coefficient: 0.437; *p* < 0.01) and old chickens (Spearman’s rank correlation coefficient: 0.434; *p* < 0.01). Additionally, in caecal samples of older chickens, a positive relationship between *Campylobacteraceae* and *Bacteroidaceae* could be observed (Spearman’s rank correlation coefficient: 0.408; *p* < 0.05), while in younger chickens, a negative Spearman’s rank correlation was found for *Campylobacteraceae* and *Comamonadaceae* (Spearman’s rank correlation coefficient: 0.501) at a significance level of *p* < 0.01. There was a negative Spearman’s rank correlation between *Campylobacteraceae* and *Erysipelotrichaceae* in samples of the young chickens, while Spearman’s rank correlation in samples of the older chickens was positive. Spearman’s rank correlation coefficients between both taxa remained low (Spearman’s rank correlation coefficient: on average ± 0.151) and insignificant independent of the chickens’ age at infection.

**TABLE 4:** Spearman’s rank correlation analysis of absolute abundance of *Campylobacteraceae* at the taxonomic level; family.

**Taxa**	**LBC-21/22 (*n* = 35)**	**LBC-70/78 (*n* = 37)**
	
	**Campylobacteraceae**	**Campylobacteraceae**
	
	**Spearman’s rank correlation coefficient**	
Comamonadaceae	–0.50098^∗∗^	–0.00922
Alcaligenaceae	0.43710^∗∗^	0.43407^∗∗^
uncultured rumen bacterium	−0.38954^∗^	–0.06161
Desulfovibrionaceae	−0.35297^∗^	0.12081
Clostridiaceae 1	0.33853^∗^	
Peptostreptococcaceae	−0.33651^∗^	–0.00529
Sphingomonadaceae	–0.27200	0.35269^∗^
Ambiguous_taxa	–0.15390	0.33483^∗^
Bacteroidaceae	–0.01011	0.40751^∗^

*Only significant correlations were plotted; ^∗^Correlation is significant at *p* < 0.05; ^∗∗^Correlation is significant at *p* < 0.01; LBC-21/22, Lohmann Brown-Classic, infection at the age of 21/22 days; LBC-70/78, Lohmann Brown-Classic, infection at the age of 70/78 days.*

### Fermentation Pattern in Caecal Chyme

The concentration of the bacterial fermentation products n-butyrate, acetate and propionate were significantly higher in older chickens compared to younger birds (Table 5). Differences in VFA concentrations between the experiments were demonstrated as being insignificant both in samples of younger chickens and in older birds (Table 6). Spearman’s rank correlation analysis between bacterial families and concentrations of VFAs demonstrated a negative relationship between *Campylobacteraceae* and concentrations of n-butyrate, acetate and propionate. The highest coefficient within bacterial families and concentrations of VFAs was seen for *Campylobacteraceae* and n-butyrate (−0.51141; *p* < 0.0001; Table 7).

**TABLE 5:** Characterisation of fermentation products (mmol/kg fresh matter) in caecal contents of chickens of different ages at time of infection.

**Group**	***N***	**Acetic acid**		**Propionic acid**		**n-Butyric acid**	
		**Mean**	**SD**	**Mean**	**SD**	**Mean**	**SD**
LBC-21/22	35	58.7^B^	28.6	3.34^B^	1.79	14.3^B^	14.6
LBC-70/78	37	92.4^A^	23.2	5.50^A^	2.72	34.7^A^	11.0

*LBC-21/22, Lohmann Brown-Classic, infection at the age of 21/22 days; LBC-70/78, Lohmann Brown-Classic, infection at the age of 70/78 days; ^*A,B*^values within a column with different superscripts differ significantly at *p* < 0.001.*

**TABLE 6:** Characterisation of the fermentation products (volatile fatty acid [VFA]) in the caecal contents of each experimental replicate.

**LBC-21/22**		**Exp 1 (*n* = 12)**		**Exp 2 (*n* = 12)**		**Exp 3 (*n* = 11)**	
**VFA**		**Mean**	**SD**	**Mean**	**SD**	**Mean**	**SD**
Acetic acid	[mmol/kg	62.3	34.8	54.7	21.1	59.3	30.4
	fresh matter]						
Propionic acid		2.96	1.18	2.95	1.36	4.17	2.50
n-Butyric acid		15.9	17.1	10.6	9.95	16.4	16.5

**LBC-70/78**		**Exp 1 (*n* = 12)**		**Exp 2 (*n* = 13)**		**Exp 3 (*n* = 12)**	
**VFA**		**Mean**	**SD**	**Mean**	**SD**	**Mean**	**SD**

Acetic acid	[mmol/kg	85.9	27.2	98.9	21.3	91.6	20.6
	fresh matter]						
Propionic acid		5.6	3.99	5.34	1.44	5.54	2.42
n-Butyric acid		32.5	13.5	34.9	9.26	36.6	10.6

*LBC-21/22, Lohmann Brown-Classic, infection at the age of 21/22 days; LBC-70/78, Lohmann Brown-Classic, infection at the age of 70/78 days; no statistically significant differences could be found between the experiments.*

**TABLE 7:** Spearman’s correlation analysis of absolute abundance of bacterial families and fermentation products in all samples.

	**Acetic acid**	**Propionic acid**	**n-Butyric acid**
	
**Taxa**	**Spearman’s rank correlation coefficient**		
Bacteroidaceae	0.22014	0.35281^∗∗^	0.24147^∗^
Campylobacteraceae	–0.45982^∗∗∗^	–0.40915^∗∗^	–0.51141^∗∗∗^
Enterococcaceae	−0.26985^∗^	–0.21472	–0.30396^∗∗^
Erysipelotrichaceae	0.44652^∗∗∗^	0.45589^∗∗∗^	0.47003^∗∗∗^
Peptostreptococcaceae	0.23533^∗^	0.21979	0.26781^∗^
Rikenellaceae	0.22481	0.14929	0.29722^∗^
Streptococcaceae	–0.20648	–0.22982	−0.23192^∗^

*Only significant correlations were plotted; ^∗^Correlation is significant at *p* < 0.05; ^∗∗^Correlation is significant at *p* < 0.01; ^∗∗∗^Correlation is significant at *p* < 0.0001.*

## Discussion

In spite of the fact that it has already be proven in chickens that microbial richness and diversity increase with age (Awad et al., 2016) with them gaining maturation (Lu et al., 2003), the microbial richness in samples of older chickens in the present study showed, contrary to expectations, no statistically significant differences compared to younger chickens.

When assessing the ratio of the concentrations of acetate to butyrate in caecal contents, noticeable differences can be seen between the chickens’ ages at infection [higher acetate:butyrate-ratio in younger chickens (2.71:1), lower ratio in older chickens (1.82:1)]. Spearman’s rank correlation analysis between *Campylobacteraceae* and the concentrations of VFA demonstrated an overall negative relationship. The highest coefficient within bacterial families and the concentrations of VFA was seen for *Campylobacteraceae* and n-butyrate. *In vitro* obtained results of a study conducted by Van Deun et al. (2008a) identified butyrate as the most successful of the examined short-chain fatty acids in being bactericidal (12.5 mM at pH 6.0) for *C. jejuni* while propionate and acetate had a bacteriostatic effect (50 mM). Nevertheless, adding of butyrate-coated micro-beads to the diet of 2-week-old broilers was unsuccessful in reducing *C. jejuni* caecal colonisation. The authors suspect behind the unsuccessful protection *in vivo* protective effects of mucous and the rapid absorption of butyrate by the enterocytes (Van Deun et al., 2008a). Different scientific working groups used Caco-2 model to investigate whether butyrate could play a protective role during *C. jejuni* infection by decreasing bacterial paracellular translocation across intestinal cell layers. Butyrate was able to protect Caco-2 cells from two major virulence mechanisms of *C. jejuni*, invasion and translocation in studies of Van Deun et al. (2008b). Additionally, Cresci et al. (2017) found that *C. jejuni* adhesion was reduced with butyrate pretreatment of Caco-2 cells, therefore showing protective effects of butyrate during *C. jejuni* infection. The authors postulated that an optimal gut microbiota composition and/or a dietary formulation with a view to enhancing butyrate levels and maintaining gut microbiota balance may influence the prevalence, incidence and outcome of *Campylobacteriosis*. Further *in vivo* investigations on potential protective effects of dietary supplemented butyrate against *Campylobacter* infection showed divergent results. Dietary supplementation with coated calcium butyrate (0.1% of the diet) had no effect on *C. jejuni* colonisation or shedding levels in experimentally infected broiler chickens (Ocejo et al., 2017). A coated butyrate-based product in a higher dosage (0.3% of the diet) was able to reduce caecal *Campylobacter* counts significantly compared to the control group during the whole fattening period. However, the observed reduction was not homogenous among the birds; some remained contaminated with caecal *Campylobacter* counts of 7 log_10_ CFU/g (Guyard-Nicodème et al., 2015).

Statistical analyses of all 216 OTUs for differences in bacterial abundance between the two age groups revealed smallest FDR-adjusted *p*-values for OTU_3 and OTU_14. OTU_ 3 represents a bacterial species that is assigned to the genus *Turicibacter* which belongs to the family *Erysipelotrichaceae.* In the present study, eight OTUs could be assigned to the family *Erysipelotrichaceae* but the family was mainly formed by the genus *Turicibacter. Turicibacter* is a well-known coloniser of livestock animals including chickens. However, little is known about this genus and its members, which may have an unknown potential to positively influence animal health (Abudabos et al., 2017). OTU_14 represents a bacterial species that is assigned to the genus *Campylobacter* which belongs to the family *Campylobacteraceae.* OTU_3 was more enriched in the older birds while OTU_14 was more enriched in the younger birds. This was consistent with the results of the quantitative bacteriological examination.

Independent of the chickens’ age at infection, Spearman’s rank correlation coefficients between both taxa remained low and insignificant, thereby indicating no relationship between the two bacterial families.

Correlation analyses of *Campylobacteraceae* with other bacterial families demonstrated no strong link in this study. A similar pattern was seen in microbiota studies of egg-laying hens (Videnska et al., 2014). In the present study, one positive association was found for the *Alcaligenaceae* family at both ages at infection. In our findings, the family *Alcaligenaceae* was formed to 100% by the genus *Parasuterella*. *Parasutterella* is a non-motile, strictly anaerobic genus which is proposed to accommodate a novel family named *Sutterellaceae* (Morotomi et al., 2011). This genus was defined as a core component of the human and mouse gut microbiota, producing succinate as a fermentative end-product while relying, as asaccharolytic genus, on amino acids such as asparagine, aspartate and serine (Ju et al., 2019). Those amino acids support its metabolic activities and physiological functions, indicating an adaptation to the gut environment, which contains readily available non-essential amino acids (Ju et al., 2019). Amino acids, in particular aspartate, glutamate, proline and serine are key carbon and energy sources for the asaccharolytic zoonotic pathogen *C. jejuni* as well (Guccione et al., 2008). Varying amino-acid concentrations in the diet of broiler chickens preferentially utilised by *C. jejuni*, such as aspartate, glutamate, proline and serine are able to influence the spread and the shedding of *C*. *jejuni* (Visscher et al., 2018). It can be hypothesised that an environment favouring *C. jejuni* metabolism should also favour growing conditions of *Parasutterella*, as their metabolism relies on similar amino acids. This would explain the positive relationship between these bacterial families.

Contrary to expectations, the factor experimental replication in the present study was discovered to contribute more to the differences of microbial composition of the samples in comparison to the factor age at infection. The dissimilarity matrix revealed higher dissimilarity of samples between experiment 1 and the other experiments. Of special interest is the higher occurrence of *Proteobacteria* in experiment 1, independent of the age of the chickens at infection, mainly consisting of the families *Campylobacteraceae* and *Alcaligenaceae* compared to the other experiments. Interestingly, the differences in VFA concentrations (n-butyrate, acetate and propionate) between the experiments were demonstrated as not being significant. This could indicate that the function of the microbiota was kept even if the microbial composition was variable between experimental replicates. The gut microbiota are immensely divers and vary interpersonal (Lozupone et al., 2012). However, the basics of microbial metabolism remain stable among the group of studied individuals because many biochemical pathways varied little among members of the microbiome (Abubucker et al., 2012). Stanley et al. (2013) pointed out that most published studies investigate changes in the microbiota of chickens in a single trial; only rarely have results been reported for replicated trials and scientific rigour requires a hypothesis to be supported by replicated results. In a previous study, they investigated microbiota changes associated with a *Clostridium perfringens* infection and noted an unexpected but important finding: there were large differences in the microbiota in the control groups between trials that were designed to be as similar as possible (same line of commercial chickens from the same hatchery, fed the same diet formulation and housed in the same facility) (Stanley et al., 2012). Therefore Stanley et al. (2013) investigated batch-to-batch variations in chicken caecal microbiota in three similar trials and equally showed that even under carefully controlled conditions, large variations in microbiota composition still occur. The authors hypothesised that this large variability is due to the colonisation by bacteria originating from the wider environment rather than predominantly from maternally derived bacteria. Thibodeau et al. (2017) examined the influence of selenium-yeast on chicken caecal microbiota in the context of colonisation by *C. jejuni* in two experimental replicates with two independent batches of chickens. They saw that bacterial community structure was significantly different between the experimental replicates and concluded that there is a need for true biological replication when studying the chicken intestinal microbiota, especially when the observed changes are subtle.

## Conclusion

Relative abundance of the families *Campylobacteraceae* and *Erysipelotrichaceae* were significantly different between younger and older chickens in all experiments without showing a link between one another. In general, correlation analyses of absolute abundance of *Campylobacteraceae* with other bacterial families revealed the strongest link to the genus *Parasutterella* and the strongest negative relationship to butyrate concentrations in caecal contents. Finally, despite performing three consecutive repetitions, the factor experimental replication was discovered to contribute more to the differences of microbial composition of the samples in comparison to the factor age at infection.

## Data Availability Statement

The datasets generated for this study can be found in the BioProject ID PRJNA557374.

## Ethics Statement

Animal experiments were performed in accordance with the German rules and regulations. The experiments were approved by the Ethics Committee of Lower Saxony for Care and Use of Laboratory Animals (LAVES) (Niedersächsisches Landesamt für Verbraucherschutz und Lebensmittelsicherheit; reference: 33.19-42502-05-15A500).

## Author Contributions

CV was the initiator of the idea and revised the manuscript. CV and JH designed the study and interpreted the data. JH carried out the experiments, collected the data with the help of CV, and wrote the manuscript. HK, BK, and CK performed the DNA extraction of the samples and prepared the samples for microbiome analyses. EG and TS carried out the microbiome analyses. KJ and JH statistically analysed the data, and compiled the figures and tables. All authors read and approved the final manuscript.

## Conflict of Interest

CK was employed by company Boehringer Ingelheim Veterinary Research Center GmbH & Co. KG. The remaining authors declare that the research was conducted in the absence of any commercial or financial relationships that could be construed as a potential conflict of interest.

## Footnotes

1 http://www.drive5.com/usearch/

2 www.r-project.org
