# Supplementary material for: Caecal Microbiota of Experimentally Campylobacter jejuni-Infected Chickens at Different Ages
Source: Front Microbiol. 2019 Oct 10;10:2303. doi: 10.3389/fmicb.2019.02303 (PMC6796544; doi:10.3389/fmicb.2019.02303)
Supplement: Supplementary file 1 [file Data_Sheet_1.docx]

Supplementary Material


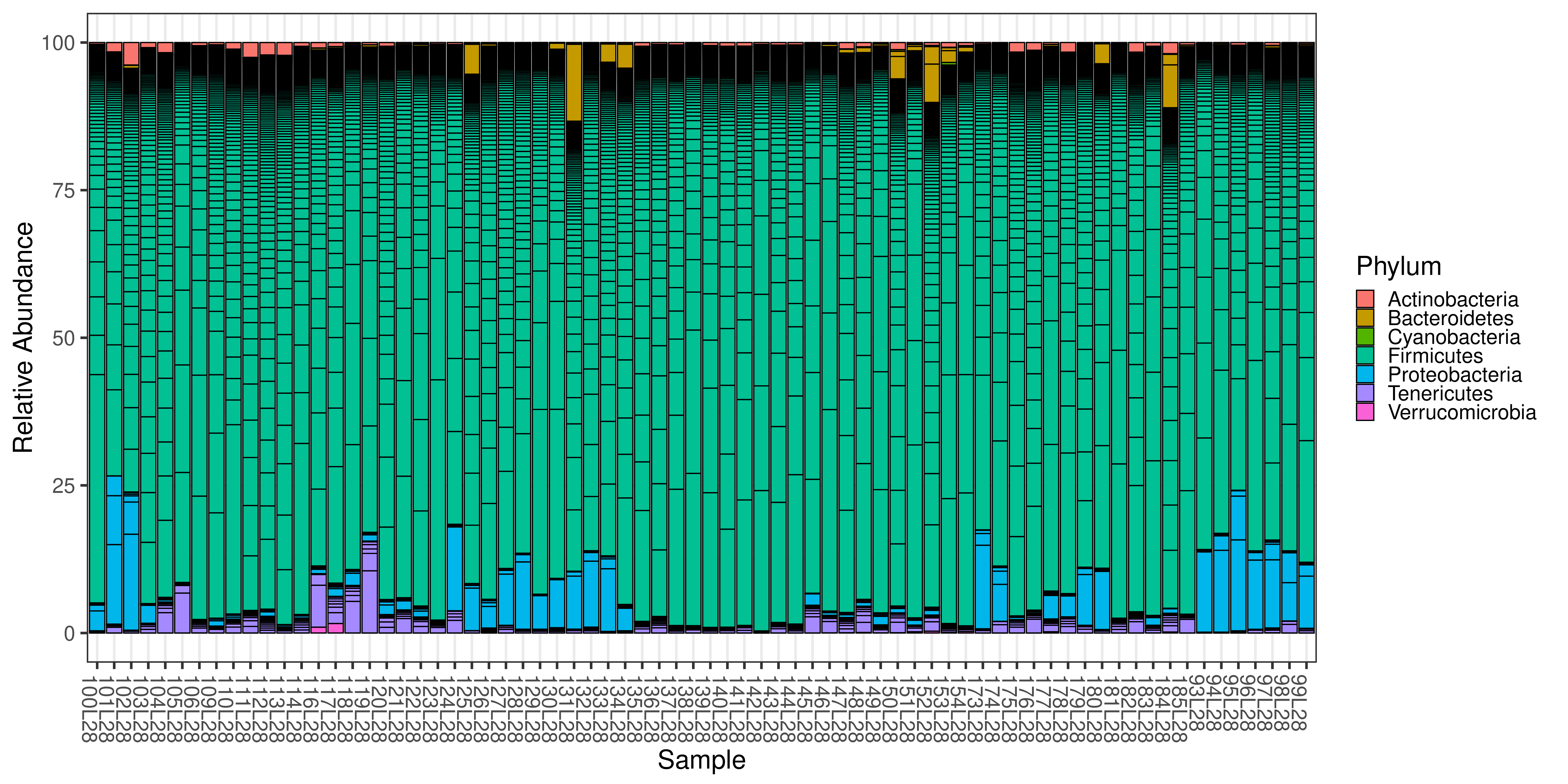


**Figure S1** Microbiota composition analysis using 16S rRNA sequencing in caecal contents of chickens. Relative abundance of bacterial phyla within all samples. Samples 93L28, 94L28, 95L28, 96L28, 173L28, 97L28, 98L28, 99L28, 100L28, 101L28, 174L28 and 102L28 belong to LBC-21/22 in experiment 1; Samples 125L28, 126L28, 127L28, 128L28, 129L28, 179L28, 130L28, 131L28, 132L28, 133L28, 134L28 and 180L28 belong to LBC-70/78 in experiment 1; Samples 103L28, 104L28, 105L28, 106L28, 109L28, 175L28, 110L28, 111L28, 112L28, 113L28, 114L28 and 176L28 belong to LBC-21/22 in experiment 2; Samples 135L28, 136L28, 137L28, 138L28, 139L28, 181L28, 185L28, 140L28, 141L28, 142L28, 143L28, 144L28 and 182L28 belong to LBC-70/78 in experiment 2; Samples 116L28, 117L28, 118L28, 119L28, 177L28, 120L28, 121L28, 122L28, 123L28, 124L28 and 178L28 belong to LBC-21/22 in experiment 3; Samples 145L28, 146L28, 147L28, 148L28, 149L28, 183L28, 150L28, 151L28, 152L28, 153L28, 154L28 and 184L28 belong to LBC-70/78 in experiment 3.

**Table S1** Comparison of bacterial relative abundance in caecal contents (at the taxonomic level, phylum) between different aged chickens at time of infection.

| Phylum | # OTUs | p-value | FDR-adjusted p-value |
| --- | --- | --- | --- |
| Firmicutes | 187 | 0.00009999 | **0.00069993** |
| Bacteroidetes | 4 | 0.00029997 | **0.00179982** |
| Tenericutes | 14 | 0.00319968 | **0.01279872** |
| Proteobacteria | 8 | 0.00469953 | **0.01879812** |
| Actinobacteria | 1 | 0.01429857 | **0.04289571** |
| Verrucomicrobia | 1 | 0.46795320 | 0.51994801 |
| Cyanobacteria | 1 | 0.51994801 | 0.51994801 |

FDR-adjusted p-values < 0.05 are written in bold.

**Table S2** Comparison of bacterial relative abundance in caecal contents (at the taxonomic level, family) between different aged chickens at time of infection.

| Family | # OTUs | p-value | FDR-adjusted p-value |
| --- | --- | --- | --- |
| Campylobacteraceae | 1 | 0.00009999 | **0.00259974** |
| Enterococcaceae | 1 | 0.00009999 | **0.00259974** |
| Erysipelotrichaceae | 8 | 0.00009999 | **0.00259974** |
| Lachnospiraceae | 49 | 0.00009999 | **0.00259974** |
| Lactobacillaceae | 2 | 0.00009999 | **0.00259974** |
| Peptostreptococcaceae | 2 | 0.00009999 | **0.00259974** |
| Ruminococcaceae | 94 | 0.00009999 | **0.00259974** |
| Streptococcaceae | 1 | 0.00019998 | **0.00499950** |
| Bacteroidaceae | 1 | 0.00069993 | **0.01609839** |
| uncultured-bacterium | 6 | 0.00139986 | **0.02939706** |
| Clostridiaceae-1 | 1 | 0.00209979 | **0.04409559** |
| Rikenellaceae | 1 | 0.00209979 | **0.04409559** |
| Coriobacteriaceae | 1 | 0.01199880 | 0.20397960 |
| uncultured-rumen-bacterium | 2 | 0.01689831 | 0.27037296 |
| Aerococcaceae | 1 | 0.02659734 | 0.39896010 |
| Enterobacteriaceae | 1 | 0.03319668 | 0.49795020 |
| Clostridiales-vadinBB60-group | 10 | 0.03629637 | 0.51369863 |
| Ambiguous_taxa | 16 | 0.03659634 | 0.51369863 |
| Family-XIII | 3 | 0.06849315 | 0.75379129 |
| Chitinophagaceae | 2 | 0.16348365 | 0.87687231 |
| Thermoanaerobacteraceae | 1 | 0.17398260 | 0.87687231 |
| Christensenellaceae | 2 | 0.20587941 | 0.91340866 |
| Veillonellaceae | 1 | 0.27607239 | 0.95740426 |
| Sphingomonadaceae | 1 | 0.31786821 | 0.95740426 |
| Defluviitaleaceae | 1 | 0.45415458 | 0.95740426 |
| Verrucomicrobiaceae | 1 | 0.47225277 | 0.95740426 |
| Phyllobacteriaceae | 1 | 0.47665233 | 0.95740426 |
| Alcaligenaceae | 1 | 0.51784822 | 0.95740426 |
| Desulfovibrionaceae | 1 | 0.56534347 | 0.95740426 |
| Peptococcaceae | 1 | 0.66563344 | 0.95740426 |
| Xanthobacteraceae | 1 | 0.73072693 | 0.95740426 |
| Comamonadaceae | 1 | 0.95740426 | 0.95740426 |

FDR-adjusted p-values < 0.05 are written in bold.

**Table S3** 28 of 216 OTUs with significant (plus an additional log fold change criterion of +/- 2) different abundance between different aged chickens at time of infection.

| log2FoldChange | pvalue | padj | OTU_ID | Phylum | Class | Order | Family | Genus |
| --- | --- | --- | --- | --- | --- | --- | --- | --- |
| 6.32948466 | 1.33E-55 | 2.85E-53 | OTU_3 | Firmicutes | Erysipelotrichia | Erysipelotrichales | Erysipelotrichaceae | Turicibacter |
| -3.89862629 | 3.46E-17 | 3.70E-15 | OTU_14 | Proteobacteria | Epsilon-proteobacteria | Campylobacterales | Campylobacteraceae | Campylobacter |
| -2.45228859 | 7.23E-15 | 5.15E-13 | OTU_12 | Firmicutes | Clostridia | Clostridiales | Ruminococcaceae | [Eubacterium] coprostanoligenes group |
| 8.94321971 | 4.24E-14 | 2.27E-12 | OTU_42 | Bacteroidetes | Bacteroidia | Bacteroidales | Bacteroidaceae | Bacteroides |
| -5.65288619 | 1.51E-12 | 6.45E-11 | OTU_85 | Firmicutes | Erysipelotrichia | Erysipelotrichales | Erysipelotrichaceae | Erysipelatoclostridium |
| 5.59167262 | 1.89E-11 | 6.73E-10 | OTU_29 | Firmicutes | Clostridia | Clostridiales | Ruminococcaceae | Ruminococcus 1 |
| -4.11016512 | 4.20E-11 | 1.28E-09 | OTU_60 | Firmicutes | Clostridia | Clostridiales | Lachnospiraceae | Ambiguous_taxa |
| 3.62911045 | 9.75E-11 | 2.61E-09 | OTU_13 | Firmicutes | Clostridia | Clostridiales | Ruminococcaceae | Subdoligranulum |
| -4.37438696 | 1.11E-10 | 2.64E-09 | OTU_54 | Firmicutes | Clostridia | Clostridiales | Ruminococcaceae | Ambiguous_taxa |
| -3.56440119 | 1.25E-10 | 2.67E-09 | OTU_63 | Firmicutes | Clostridia | Clostridiales | Ruminococcaceae | Anaerotruncus |
| -2.42026925 | 3.16E-10 | 6.14E-09 | OTU_69 | Firmicutes | Clostridia | Clostridiales | Ruminococcaceae | Ambiguous_taxa |
| -2.85982582 | 7.52E-07 | 1.07E-05 | OTU_126 | Firmicutes | Bacilli | Lactobacillales | Streptococcaceae | Streptococcus |
| 2.51176478 | 1.36E-05 | 0.00018195 | OTU_162 | Firmicutes | Clostridia | Clostridiales | Lachnospiraceae | Blautia |
| 2.12653731 | 2.07E-05 | 0.00023351 | OTU_297 | Firmicutes | Clostridia | Clostridiales | Lachnospiraceae | Ambiguous_taxa |
| -2.31443319 | 2.59E-05 | 0.00027756 | OTU_437 | Firmicutes | Clostridia | Clostridiales | Ruminococcaceae | [Eubacterium] coprostanoligenes group |
| 2.41932537 | 4.74E-05 | 0.00046096 | OTU_168 | Firmicutes | Clostridia | Clostridiales | Lachnospiraceae | Ambiguous_taxa |
| -2.92864417 | 8.12E-05 | 0.00066812 | OTU_17 | Tenericutes | Mollicutes | Mollicutes RF9 | uncultured bacterium | uncultured bacterium |
| -2.40548313 | 0.00027534 | 0.00196407 | OTU_195 | Firmicutes | Erysipelotrichia | Erysipelotrichales | Erysipelotrichaceae | Erysipelatoclostridium |
| -3.61069256 | 0.00042458 | 0.00283938 | OTU_200 | Firmicutes | Clostridia | Clostridiales | Peptostreptococcaceae | Romboutsia |
| -2.68020319 | 0.00056928 | 0.00348075 | OTU_304 | Firmicutes | Clostridia | Clostridiales | Ruminococcaceae | Flavonifractor |
| -3.46089619 | 0.00119331 | 0.00654788 | OTU_209 | Firmicutes | Clostridia | Clostridiales | Ambiguous_taxa | Ambiguous_taxa |
| 2.17733665 | 0.00244336 | 0.01244948 | OTU_120 | Tenericutes | Mollicutes | Mollicutes RF9 | uncultured rumen bacterium | uncultured rumen bacterium |
| 2.02714231 | 0.0026292 | 0.01308483 | OTU_91 | Firmicutes | Clostridia | Clostridiales | Clostridiales vadinBB60 group | uncultured bacterium |
| -2.18237298 | 0.00297871 | 0.01392541 | OTU_189 | Firmicutes | Clostridia | Clostridiales | Lachnospiraceae | Tyzzerella 3 |
| 2.56266896 | 0.00299331 | 0.01392541 | OTU_93 | Tenericutes | Mollicutes | Mollicutes RF9 | uncultured bacterium | uncultured bacterium |
| -2.03361244 | 0.01162044 | 0.04434364 | OTU_187 | Firmicutes | Bacilli | Lactobacillales | Enterococcaceae | Enterococcus |
| -3.94357235 | 0.01181116 | 0.04434364 | OTU_149 | Firmicutes | Clostridia | Clostridiales | Lachnospiraceae | Ambiguous_taxa |
| 2.39813291 | 0.0132418 | 0.04885768 | OTU_219 | Firmicutes | Erysipelotrichia | Erysipelotrichales | Erysipelotrichaceae | Ambiguous_taxa |
